# A qualitative study to refine and finalize the MedManageSCI prototype: A web-based toolkit to support medication self-management in adults with spinal cord injury/dysfunction

**DOI:** 10.1371/journal.pdig.0001054

**Published:** 2025-10-22

**Authors:** Lauren Cadel, Rasha El-Kotob, Sander L. Hitzig, Lisa M. McCarthy, Shoshana Hahn-Goldberg, Tanya L. Packer, Tejal Patel, Chester H. Ho, Stephanie R. Cimino, Aisha K. Lofters, Sara J. T. Guilcher

**Affiliations:** 1 Leslie Dan Faculty of Pharmacy, University of Toronto, Toronto, Canada; 2 Institute for Better Health, Trillium Health Partners, Mississauga, Canada; 3 St. John’s Rehab Research Program, Sunnybrook Research Institute, Sunnybrook Health Sciences Centre, Toronto, Canada; 4 Rehabilitation Sciences Institute, Temerty Faculty of Medicine, University of Toronto, Toronto, Canada; 5 Department of Occupational Science and Occupational Therapy, Temerty Faculty of Medicine, University of Toronto, Toronto, Canada; 6 Dalla Lana School of Public Health, University of Toronto, Toronto, ON, Canada; 7 Department of Family and Community Medicine, University of Toronto, Toronto, Canada; 8 Women’s College Research Institute, Toronto, Canada; 9 Schlegel-UW Research Institute of Aging, Waterloo, Canada; 10 OpenLab, University Health Network, Toronto, Canada; 11 School of Health Administration, Dalhousie University, Halifax, Canada; 12 Department of Nursing, Umeå University, Umeå, Sweden; 13 School of Pharmacy, University of Waterloo, Kitchener, Canada; 14 Division of Physical Medicine & Rehabilitation, Department of Clinical Neurosciences, Foothills Medical Centre, Calgary, Canada; 15 Lawson Research Institute, St. Joseph’s Health Care London, London, Canada; 16 Institute of Health Policy, Management and Evaluation, University of Toronto, Toronto, Canada; 17 Department of Physical Therapy, Temerty Faculty of Medicine, University of Toronto, Toronto, Canada; Jos University Teaching Hospital, NIGERIA

## Abstract

Adults with spinal cord injury/ dysfunction (SCI/D) commonly take multiple medications for a variety of secondary conditions, and have described challenges with medication self-management. To help support medication self-management, a web-based toolkit, MedManageSCI, was co-designed by our team of researchers and adults with SCI/D, caregivers, and healthcare providers (www.medmanagesci.ca). Together, we co-developed the content areas to include in MedManageSCI, along with the design and brand considerations, to create an initial prototype of the toolkit. To finalize the prototype prior to implementation, the primary objective of this qualitative study was to further refine MedManageSCI by examining the clarity, comprehensiveness, relevance, and delivery of the toolkit modules. Cognitive interviews were conducted virtually between July 2024 and September 2024 with adults with SCI/D (N = 16). A concurrent verbal probing approach using scripted and spontaneous probes was followed. Data were coded using a pre-established coding matrix that aligned with the scripted probes. Participants provided 193 specific modifications to improve the clarity, comprehensiveness, relevance, or delivery of the MedManageSCI toolkit, which were categorized as: Comprehension, Design, and Web-based Delivery. The Comprehension category contained three subcategories: Written Refinements, Ensuring Accessibility, and Revamping Resources. The Design category contained three subcategories: Formatting Content, Streamlining Function, and Enhancing Visuals. Participants perceived the website as an ideal way to deliver the toolkit, noting several benefits of a web-based delivery in comparison to a paper-based toolkit. Overall, participants found the modules to be comprehensive and highly relevant. Further, we discuss the application of cognitive interviews for further refining the MedManageSCI prototype, recommendations to improve the comprehensibility, and the advantages of a web-based toolkit for the SCI/D population. Involving individuals with SCI/D in the development and refinement of self-management materials will help ensure that the content and resources are tailored and appropriate; thereby elevating its likelihood of uptake and dissemination during implementation.

## Author summary

Adults with spinal cord injury/dysfunction (SCI/D) often take a number of medications to manage secondary conditions that occur post-injury. There were few resources that comprehensively supported individuals with SCI/D in managing their medications, so our team co-designed the prototype of a web-based toolkit called MedManageSCI. Before making the toolkit publicly available online, we wanted to refine and finalize the MedManageSCI prototype. We conducted interviews with 16 adults with SCI/D from across Canada to examine the clarity, comprehensiveness, relevance, and delivery of the toolkit modules. We received feedback to improve the clarity of information and design elements. Revisions to the MedManageSCI prototype are now complete and the website is publicly available for use (www.medmanagesci.ca). Overall, participants shared both positive and constructive feedback on the web-based MedManageSCI toolkit. Our study provides insights into the benefits of including end-users in the prototype development and refinement process. Next steps of this research include examining the feasibility, acceptability, and appropriateness of MedManageSCI.

## Introduction

Spinal cord injury/dysfunction (SCI/D) affects over 15 million people internationally [1]. SCI/D occurs when the spinal cord is damaged due to a traumatic or non-traumatic event [2], resulting in temporary or permanent disruptions to an individual’s autonomic, sensory, and motor function [3]. Consequently, adults with SCI/D frequently experience secondary conditions, which may be acute, chronic, or episodic in nature. Common secondary conditions include, but are not limited to: pain, spasticity, urinary tract infections, pressure sores, neurogenic bladder and bowel, osteoporosis, and mental health conditions [4,5]. Secondary conditions can result in hospitalization [6], and many may also impact an individual’s ability to work, and participate in social and daily life activities [7–9].

Medications are one method of preventing, treating, managing, or minimizing the impact of secondary conditions. Adults with SCI/D commonly take multiple medications, including prescription medications, over-the-counter medications, and natural health products [10–14]. For instance, a person with SCI/D may be prescribed an anti-depressant to manage depression and/or neuropathic pain, take over-the-counter ibuprofen for joint pain, and take daily doses of Vitamin D supplements to support bone health. While often appropriate for specific symptom relief, the use of multiple medications, often referred to as polypharmacy, can lead to problems, such as adverse reactions. Increased cost and healthcare utilization, for example emergency department visits, may also occur [15]. In addition to these risks, adults with SCI/D report challenges and concerns taking multiple medications, including dealing with medication side effects and safety, understanding how to best manage daily medication taking, playing an active role in medication-related decisions, and navigating a changing identity that includes taking medications [16].

Given the high prevalence of multiple medication use among individuals with SCI/D and the associated challenges, medication self-management is of key importance whether medication use is temporary for acute conditions such as for urinary tract infections, ongoing for chronic conditions such as neuropathic pain, or as needed for episodic conditions such as muscle pain. Medication self-management is defined as the everyday tasks, skills, and behaviours needed to manage the physical, social, and cognitive aspects of taking, or choosing not to take medications [17]. In comparison to other populations that commonly take multiple medications, adults with SCI/D may have unique medication needs. Individuals with SCI/D may experience physical impairments post-injury, which can make medication self-management tasks and skills more complex. Currently, there are few medication self-management tools and supports for the SCI/D population and of those that exist, most do not include all aspects of medication self-management [17].

To help support medication self-management among adults with SCI/D, a prototype of a toolkit, MedManageSCI, was co-designed by our research team, adults with SCI/D, caregivers, and healthcare providers (www.medmanagesci.ca) [18]. MedManageSCI is a web-based toolkit that aims to provide individuals with SCI/D with the knowledge, tools, and confidence to seamlessly integrate medications into their everyday lives and promote their overall health and well-being. To our knowledge, MedManageSCI is the first digital toolkit co-created with the end-users to specifically and comprehensively address medication self-management for adults with SCI/D, thus providing a unique resource for this population. The co-design process was guided by the Good Things Foundation Pathfinder Model [19]. It focused on the content areas to include in MedManageSCI and the design and brand considerations. MedManageSCI was designed to be comprehensive in covering the core tasks (medical, emotional, and role management) and skills (problem solving, decision making, seeking formal and informal supports, goal setting, and engaging in activities) of medication self-management for adults with SCI/D [20]. This web-based toolkit consists of nine modules to provide the SCI/D community with information, practical tips, and strategies for effective medication management. The modules cover the following topics: background information about medication self-management, general medication information, self-advocacy, communication with healthcare providers, practical tips and strategies for medication management, safety and side effects of medications, access to medications, supplies and services, peer support, and managing expectations related to medication self-management post-injury. Each module consists of written information and visual content in the form of infographics, videos, and pictures.

To ensure the content and resources contained within the toolkit are easy to read, understandable, and culturally appropriate, Hempel and colleagues recommend seeking feedback from the end users [21]. As such, during the development of the MedManageSCI prototype and prior to implementation (making the website publicly available), it was important to seek in-depth feedback on the toolkit to ensure the content was comprehensible to the end-users, adults with SCI/D [21]. Therefore, to finalize our prototype, the primary objective of this study was to further refine the MedManageSCI toolkit by examining the clarity, comprehensiveness, relevance, and delivery (i.e., content and design optimization) of the toolkit modules. The secondary objective was to explore the participants’ reactions to the toolkit prototype and opinions on the design.

## Materials and methods

### Study design

**Fig 1:**
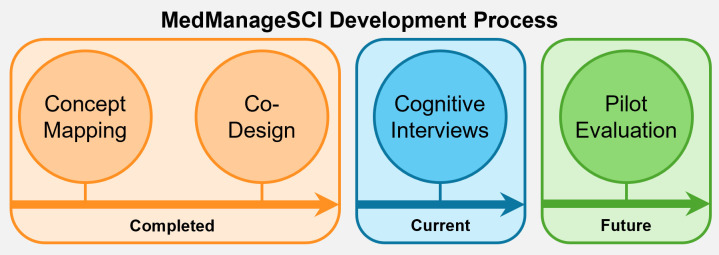
MedManageSCI development process.

This qualitative study was situated within a larger, mixed methods study, where the overall objective was to co-develop, revise, and evaluate a medication self-management toolkit for adults with SCI/D (see Fig 1 for development process). First, we conducted a concept mapping study with adults with SCI/D, caregivers, and healthcare providers to explore and prioritize content areas to include in the toolkit [22]. We then co-designed a prototype of the MedManageSCI toolkit, which focused on the content areas to include in the toolkit, design elements, and brand considerations [18]. Using input from individuals with SCI/D, our research team (LC, RE, SJTG) developed the written content for the modules based on research evidence, best practices, guidelines, and lived experiences. This content was reviewed by members of the research team and experts in the field but was not reviewed by the end-users. Herein, we report on the next phase of this research that involved a qualitative approach to further refine the toolkit prototype to ensure the modules were clear, comprehensive, relevant, and delivered in an appropriate manner from the perspective of adults with SCI/D. The research question guiding this study was: *how do adults with SCI/D perceive and understand the content and design of MedManageSCI?* The Standards for Reporting Qualitative Research were followed in reporting this research (see S1 File) [23].

### Research paradigm and reflexivity

The overarching research was grounded in pragmatism [24], which accepts there can be a single or multiple truths and realities [25]. We believed that participants’ realities and knowledge would be based on their habits, beliefs, prior experiences, and social interactions [26].

The researchers conducting data collection and analysis (LC, MSc and RE, PhD) have qualitative experience and were overseen by a senior member of the team (SJTG, PhD). The researchers (LC, RE) had a prior relationship with some participants due to involvement in previous phases of this research. We believe this strengthened the quality of the data because participants were able to provide honest and constructive feedback [27]. The core research team (SJTG, LC, RE) met regularly to discuss recruitment, data collection, and analysis and during these discussions, the researchers reflected on how their prior experience and positionality may have shaped their perspectives of the findings. These discussions were captured through meeting notes.

### Participants and recruitment

Adults with SCI/D were included as study participants as the targeted end-users of MedManageSCI. For inclusion, participants were required to have either traumatic or non-traumatic injuries, be at least three months post-injury, live in Canada, be able to read and communicate in English, and be at least 18 years of age. Purposive, convenience, and snowball sampling strategies were used for recruitment. Specifically, we contacted participants from previous phases of this research, distributed information through several partner organizations and our research teams’ networks, and posted flyers on social media. Participants were able, but not required, to have participated in the first phase of this study, the initial co-design of the toolkit.

### Data collection

Data were collected through cognitive interviews conducted by two members of the research team (LC and RE) between July 2024 and September 2024. Cognitive interviews are used to explore how individuals understand information [28], where qualitative interviews are often used to explore how individuals experience and describe a phenomena of interest [29]. Cognitive interviews are advantageous for a number of reasons, including their ability to provide insight into how individuals comprehend information, retrieve information, and process decisions [30]. As such, we undertook cognitive interviews to explore how participants understood the content contained in MedManageSCI.

The interviews were conducted virtually using Zoom. Only the interviewer (research team member) and participant were present during the interview. In terms of process, the interviewer described the plan and objectives of the cognitive interview, answered any questions the participant had, and shared their screen with the MedManageSCI website prototype. All participants started on the toolkit landing page and were then guided to the pre-selected modules by the interviewer. A concurrent verbal probing approach using scripted and spontaneous probes was followed. The scripted probes were outlined in an interview guide and focused on the toolkit content and design optimization (see S2 File). More specifically, following each section of the module, the interviewer asked the participant what thoughts came to mind as they read the information, if any words or phrases were unclear or confusing, to explain the section in their own words, to describe their thoughts on the length of the module, and to discuss if they thought the module would be useful to themselves or to someone else with SCI/D. We also explored the participants’ initial reactions to the toolkit, opinions on the design, thoughts about accessing the toolkit on a website versus other methods, and any additional feedback to improve the toolkit.

All interviews were approximately 60 minutes in length and audio recorded. Participants reviewed parts of the MedManageSCI prototype and provided feedback on the toolkit modules. The number of modules reviewed during the interview varied, with a range between 0.5 and 3 modules. This was because all cognitive interviews were the same duration of time (60 minutes); however, the length of the modules was not consistent, and the amount of feedback provided differed across participants. Interviews were conducted until a minimum of two individuals had reviewed each module (see S3 File). This was driven by feasibility of recruitment and the concept of information power, which specifies that the more information the sample holds, a smaller number of participants is required [31]. Given the specific aim of the study paired with participants with highly specific characteristics, this sample was deemed as sufficient. Each participant provided focused, highly relevant, and in-depth information relative to the purpose of the research, thus requiring fewer participants to achieve information power [31]. Additionally, many of the recommendations provided by participants were applicable across modules, which increased the relevant feedback obtained per module. Detailed notes were taken by the interviewer during the interview to support analysis.

### Data analysis

Data analysis followed an iterative process that began after the first interview. Based on the scripted prompts in the interview guide, a deductive coding matrix was used to organize the data (see S4 File). Following each interview, the interviewer listened to the audio-recording and deductively coded the data directly into the coding matrix. They also reviewed their detailed notes and added any relevant and/or missing information. The purpose of the analysis was to identify content to be revised, expanded, or removed based on the participants’ feedback on the clarity, comprehensiveness, relevance, delivery, and design of the module content. Once the data were recorded in the matrix, a second researcher reviewed the information for accuracy and completeness by reading the matrix and listening to the audio recording when necessary. Intercoder agreement was not assessed because the purpose of the secondary coder was to expand the analysis. However, if there were any discrepancies in how or where the data were recorded, the two researchers reached consensus through discussion. Each researcher kept detailed notes and reflexive memos that were used to guide these discussions.

All suggested revisions were transferred to a new document for categorization and tracking. Each revision was descriptively and inductively coded as one of the following: Written, Accessibility, Resource, Format, Layout, Function, or Visual, and later categorized as Comprehension, Design, or Web-based Delivery. During the analysis process, the researchers (LC, RE) examined both dominant trends and discoveries. Dominant trends are comments or feedback that occur repeatedly across participant interviews, while discoveries may not be recurring but are important as they can harm the data quality [32]. Dominant trends were addressed by revising the toolkit, while discoveries were first discussed by the research team before making any changes. If any suggested revisions could not be made, clear documentation was recorded with the rationale.

### Ethics

All participants provided written consent prior to taking part in this study. This study received ethics approval from the Research Ethics Boards of the University of Toronto (#42195) and the University of Alberta (#Pro00121103). All research was carried out in compliance with the Declaration of Helsinki.

## Results

### Participant characteristics

Sixteen individuals participated in this study (see Table 1 for participant characteristics). No individuals dropped out of the study; one individual consented to participate but did not complete an interview due to scheduling difficulties. Over half of the participants reported to be female and identified as cis-gendered women (n = 10, 62.5%), while the other six participants (37.5%) were male and identified as cis-gendered men. Participants were aged between 28 and 72, with a median age of 60 (IQR 42–65). Twelve (75%) had traumatic injuries and 4 (25%) had non-traumatic injuries. The median time post-injury was 16.5 years (IQR 8.5-26.5). The majority of participants identified as White (n = 11, 68.8%), with two identifying as Black (12.5%), and three as other races (18.8%). All the participants were taking medication; five (31.3%) had a regimen between 1 and 4 medications, six (37.5%) had a regimen between 5 and 9 medications, and five (31.3%) had a regimen of 10 or more medications. These regimens consisted of prescription medications, over-the-counter medications, and natural health products. Some medications were taken routinely, while others were taken as needed.

**Table 1:** Participant demographic and injury characteristics.

Characteristic	Number (%)
**Province**	
Ontario	9 (56.3%)
Alberta	2 (12.5%)
British Columbia	4 (25.0%)
Other	1 (6.3%)
**Age**	
18-39	2 (12.5%)
40-49	2 (12.5%)
50-59	4 (25.0%)
60+	7 (43.8%)
**Sex**	
Male	6 (37.5%)
Female	10 (62.5%)
**Gender**	
Man	6 (37.5%)
Woman	10 (62.5%)
**Race**	
White	11 (68.8%)
Black	2 (12.5%)
Other	3 (18.8%)
**Marital Status**	
Married/ Domestic Partnership	10 (62.5%)
Separated/ Divorced	2 (12.5%)
Never Married	4 (25.0%)
**Area of Residence**	
Rural Area	2 (12.5%)
Small Town/ Village	2 (12.5%)
Town/ Small City	5 (31.3%)
Big City	7 (43.8%)
**Household Income**	
$15,000 - $24,999	2 (12.5%)
$25,000 - $34,999	2 (12.5%)
$35,000 - $49,999	3 (18.8%)
$50,000 - $74,999	3 (18.8%)
$75,000 - $99,999	0 (0.0%)
$100,000+	3 (18.8%)
Prefer not to say	3 (18.8%)
**Birth Country**	
Canada	14 (87.5%)
Other	2 (12.5%)
**Injury Type**	
Traumatic	12 (75.0%)
Non-traumatic	4 (25.0%)
**Level of Injury**	
Cervical	8 (50.0%)
Thoracic	4 (25.0%)
Lumbar/ Sacral	2 (12.5%)
Unknown	2 (12.5%)
Completeness of Injury*	
Complete	5 (31.3%)
Incomplete	11 (68.8%)
**Years Post Injury**	
0-4	2 (12.5%)
5-14	4 (25.0%)
15-24	4 (25.0%)
25-34	2 (12.5%)
35+	2 (12.5%)
Not reported	2 (12.5%)
**Cause of Injury**	
Transport	7 (43.8%)
Sports/ Leisure	2 (12.5%)
Other	7 (43.8%)
ASIA Impairment**	
A – Complete	5 (31.3%)
B – Incomplete, Sensory Preserved	3 (18.8%)
C – Incomplete, Motor Non-Functional	3 (18.8%)
D – Incomplete, Motor Functional	3 (18.8%)
Unknown	2 (12.5%)
**Medication Use**	
1-4	5 (31.3%)
5-9	6 (37.5%)
10+	5 (31.3%)

*Completeness of injury refers to the preservation or lack of preservation of motor and sensory function below the neurological level of injury [33].

**ASIA Impairment refers to a standardized evaluation used to assess the sensory and motor levels which were affected by the SCI/D [33].

### MedManageSCI reflections and refinements

Overall, participants had positive reactions to the MedManageSCI prototype, with the majority stating that they would recommend the toolkit to other individuals with SCI/D. All modules were reviewed by a minimum of two participants. Overall, the modules were considered relevant to their medication management needs. Some participants noted that various topic areas would be more applicable to those who were newly injured or who were having difficulties with specific aspects of medication management. The information contained within the modules was described as clear and comprehensive. The participants identified 193 specific modifications to improve the MedManageSCI toolkit, which were categorized as: Comprehension, Design, and Web-based Delivery (see Table 2 for categories, subcategories, and examples). The Comprehension and Design categories contained the majority of feedback provided by participants.

**Table 2:** Categories and subcategories of revisions with examples.

Category	Subcategory	Example Problem/ Feedback	Example Revision/ Action Taken
Comprehension	Written Refinements	**Module 1: Background Information about Medication Self-Management** **Original:** “Emotional management includes dealing with different emotions or feelings that are associated with taking and managing medications.” **Problem:** Certain medications can alter individuals’ moods as a side effect, but this is not clear in the definition of emotional management.	**Module 1: Background Information about Medication Self-Management** **Revision:** “Emotional management includes dealing with different emotions or feelings that are associated with taking and managing medications and their side effects.” **Action Taken:** Updated the definition of emotional management to include medication side effects.
	Ensuring Accessibility	**Module 8: Peer Connections and Support** **Problem:** Vague reference to peer programs that may exist, but user is not able to access them/ still has to search for information on their own.	**Module 8: Peer Connections and Support** **Action Taken:** Addition of clickable links that take users directly to source of programs and/or information.
	Revamping Resources	**Module 2: General Medication Education and Awareness** **Problem:** Redundancy within the healthcare provider information sheet, as description of the healthcare provider and when to contact are repetitive.	**Module 2: General Medication Education and Awareness** **Action Taken:** Healthcare provider information sheet was simplified to reduce redundancy. The description of the healthcare provider was kept on the website and the downloadable resource was just used for tracking contact information.
Design	Formatting Content	**Multiple Modules** **Problem:** Some of the modules start with videos; they have no introduction to give the user context of what the video will be about.	**Multiple Modules** **Action Taken:** Re-arranged the information within the modules to ensure all videos have a text introduction.
	Streamlining Function	**Module 3: Self-Reflections and Advocacy** **Problem:** The written content in Module 3 refers the reader to Module 5 and Module 9 if they would like more information on the topic; these modules should be linked to help the user easily navigate to the referenced sections.	**Module 3: Self-Reflections and Advocacy** **Action Taken:** Modules 5 and 9 were linked to help with easier navigation to relevant information.
	Enhancing Visuals	**Module 2: General Medication Education and Awareness** ***Infographic (Routes of Medication Administration)*** **Problem:** The icon of the tongue for sublingual/buccal administration is not clear; it should clearly visualize that the medication goes under the tongue and not just in the mouth. It should be visually distinct from oral administration.	**Module 2: General Medication Education and Awareness** **Action Taken:** The icon of the tongue was changed to show it lifted in the mouth, with an arrow indicating that the medication goes underneath it.
Web-based Delivery	N/A	**Problem:** There may be users who have limited access to technology or who would prefer a paper-based toolkit.	**Action Taken:** Not applicable; future work may involve translating the online toolkit into a PDF.

### Comprehension

The Comprehension category contained three subcategories: Written Refinements, Ensuring Accessibility, and Revamping Resources. Most recommendations to improve the clarity of information were specific to the written content. Within the Written Refinements subcategory, revisions were made to correct proofreading errors (e.g., spelling and punctuation), minimize repetition of words or information, simplify the information by using lay words or adding examples, adding practical notes and suggestions, revising sentence structure to emphasize key points, and expanding on certain details (e.g., aging, pneumonia) to emphasize the connection to medication use. For example, the Managing Expectations and Adapting to Change module contained a section on possible changes to medication management as someone ages with SCI/D. Within this section, participants expressed some confusion with the information on muscle decline and fatigue that often occurs with aging and how that may impact medication management. We added a statement clarifying that muscle decline and fatigue may change an individuals’ functional abilities and therefore, they may require new or adapted supports. We also added potential strategies that individuals could engage in if experiencing age-related factors impacting their medication management. Recommendations within this category that were not addressed included: adding ‘spinal cord injury/ dysfunction’ to all module titles, explaining that brand name and generic medications do not have the same level of effectiveness, adding a key messages box to each page, and adding a module on wound healing. We did not address these suggestions because the titles had been decided on in consultation with a working group, the recommendation was not supported by scientific evidence, the design of the website did not support the boxes, and wound healing is not specific to medication management.

The Ensuring Accessibility subcategory contained recommendations and revisions to improve the user’s ability to access the information and resources, as well as general accessibility considerations. For example, including links to other websites with relevant information and ensuring all pictures had alternative text for people who are blind or partially sighted. A key recommendation within Accessibility was to have the toolkit available in multiple languages. Unfortunately, within the scope of this research, this was not feasible, but it is a consideration for future work.

The Revamping Resources subcategory contained all recommendations and revisions to the downloadable resources contained within the toolkit, some of which aligned with other subcategories (e.g., written, visual, format, layout). For example, one downloadable resource contained a table with information on medication considerations before, during, and after travel. However, within the table, it was not clear to participants where the differentiation between before, during, and after occurred. We revised the formatting of the table by adding spacing and bold line borders between the sections to visually differentiate them. This category also included creating new downloadable resources (e.g., common questions to ask your healthcare provider about non-prescription medications) when recommended by participants.

### Design

The Design category contained three subcategories: Formatting Content, Streamlining Function, and Enhancing Visuals. Formatting Content included revisions to the visual presentation and organization of information in the modules, including adding introductions to the pages within each module, rearranging content within the module, creating subheadings to help with module navigation, bolding words or terms to add emphasis, adding line-by-line colour coding to the tables, and adding bullets and line spacing to breakup large sections of text. Streamlining Function included adding more links between pages within the toolkit, as well as to external resources, and adding terms to the glossary so they had linked definitions available to the user. Enhancing Visuals included revisions to the infographics, videos, and pictures contained on the website. For example, infographics were revised to improve the clarity of the visual messaging, videos were revised to change the colour contrast of items and words against the background, and pictures were replaced to increase their relevance and representativeness of the population (e.g., more up to date wheelchairs). Recommendations within the Design category that were not addressed included: combining and rearranging the order of some modules, making the font size of the headings smaller and the body text larger, and changing the colour of the subheadings. These recommendations were not addressed because the organization of the modules was based on our co-design study and the design elements (font size and colours) had been finalized with our website development company and could not be changed. Additionally, participants recommended including more diversity in the videos in terms of injury level by showing characters with different mobility aids (i.e., manual wheelchair, power wheelchair, walker). We were unable to fully address this recommendation due to limitations within the video software (walkers were not available).

### Web-based delivery

Participants thought a website was an ideal way to deliver the toolkit modules, noting several benefits of a web-based delivery in comparison to a paper-based toolkit. Given the nature of how the cognitive interviews were conducted, with the researcher sharing their screen with the participant on Zoom, all participants viewed the toolkit on a computer, rather than on a tablet or mobile phone. The benefits of a web-based delivery included having a wider reach, being easier to update, being unable to lose or misplace it, and being easier to refer to during appointments with healthcare providers. Participants mentioned that it would be beneficial to also offer a paper-based option, as some individuals may have limited access to technology or may prefer a physical copy based on their learning style.

### Recommendations for Web-Based Toolkit Prototype Development

In Table 3 (available for download in S5 File), we have outlined a checklist of recommendations for developing web-based toolkits, which is based on the feedback we received to improve the comprehension, design, and delivery of MedManageSCI. This checklist includes recommendations to ensure the written content is clear, comprehensive, and relevant to the end-users, the visual content supplements the written information and is accessible, and the delivery of the toolkit meets the needs of the end-users.

**Table 3:** Checklist of recommendations for web-based toolkit prototype development.

Completed?	Recommendations for web-based toolkits
☐	Complete multiple rounds of revisions to the written content: • Research team members • Experts in the field outside of the direct research team • End-users • Other individuals not directly involved in the creation of the content
☐	Read written content out loud to team members or end-users to catch repetitive words or phrases
☐	Use headings and subheadings to organize the information on the website pages and within the modules
☐	Add bullets and spacing to break-up larger sections of text
☐	Use accessible language and check literacy levels of the written content: • If literacy level is high (based on readability scores), add practical examples and visuals (e.g., infographics, videos) to supplement the written information
☐	Add a glossary so frequently used words and complex terms or phrases have a definition linked to them
☐	Emphasize key points through formatting – add icons, bold and/or underline words
☐	Include direct links to references and other similar or relevant resources
☐	Ensure all downloadable resources are fillable (e.g., they can be completed digitally on a computer, tablet, or mobile phone, or they can be printed and written on)
☐	Use line-by-line colour coding in larger tables (e.g., those with multiple columns and rows)
☐	Use colour contrast checkers to ensure colour combinations meet accessibility requirements
☐	Involve end-users in the selection of pictures/images for the toolkit to ensure all pictures are representative of the population
☐	Include diversity in the selected images (e.g., differing ages, genders, levels of injury, types of mobility devices)
☐	Include alternative text for all pictures
☐	Ensure the website meets web accessibility standards
☐	Use a design that allows for the creation of a paper-based version
☐	Consider having the information available in multiple languages
☐	Seek ongoing feedback from the end-users throughout the process

## Discussion

In this qualitative study, we conducted cognitive interviews among 16 adults with SCI/D to further refine and finalize the MedManageSCI prototype by examining content and design optimization recommendations. Overall, participants reflected positively on the toolkit, but also provided key recommendations for improving MedManageSCI, which were categorized into three areas: Comprehension, Design, and Web-based Delivery. We will discuss the application of cognitive interviews for further refining toolkit content, recommendations made by participants to improve the comprehensibility of information in the modules, advantages of a web-based toolkit for adults with SCI/D, and considerations around implementation and dissemination of MedManageSCI.

Cognitive interviews are typically used for questionnaire development to ensure respondents interpret the items as intended [34]. They identify words and phrases that respondents may misunderstand or misinterpret, and determine how respondents retrieve and process information [30]. However, as demonstrated in this study, cognitive interviews can be useful in contexts beyond questionnaire development for ensuring the comprehensibility and accessibility of information. For instance, in this study, we used cognitive interviews to revise content contained within an educational resource prior to implementation. The use of cognitive interviews for revising educational material and toolkit content has been previously conducted [35–38]. For example, Mills and Haga conducted qualitative cognitive interviews among pharmacists and members of the public to assess the understandability of utility of a pharmacogenetic educational toolkit, PGx [37]. In doing so, revisions were made to the toolkit to reduce the amount of text, improve the understandability of information, replace images, and enhance the formatting of text to draw attention to key points. Based on the results from our cognitive interviews, we similarly revised the MedManageSCI toolkit to improve how the information was understood by participants, replace images that were not representative of the SCI/D community, and revise the formatting to emphasize key messages. Mills and Haga also discussed the importance of utilizing a participatory design in the development of their educational materials to support its comprehension, utilization, and effectiveness [37], which we found to be a key step in the process of creating the MedManageSCI prototype [18]. Ultimately, cognitive interviews offer an in-depth and structured approach to obtaining feedback on educational material and toolkit content to ensure the accessibility and comprehensibility of the presented information.

The majority of feedback obtained from our cognitive interviews was categorized as Comprehension, with recommendations to improve the written content, accessibility of information, and resources contained within the toolkit. Participants suggested ways to improve the written content by correcting proofreading errors, reducing repetition of words or phrases, simplifying the information by adding practical examples, emphasizing key points, and expanding on details where the connection to medication was not clear. Similar results were identified by Henriksen and colleagues during their development and validation of MedHipPro-Q, a questionnaire assessing medication management among patients with hip fracture [39]. Cognitive interviews were conducted among 23 participants to identify potential problems and ensure the content was being interpreted as intended. Two themes, Representativeness and Presentation, were identified as problems. The subthemes and categories within Henriksen’s Presentation aligned with our results. More specifically, Henriksen et al. identified the following categories: proofreading errors, ambiguous terms and expressions, complicated syntax, passive voice, inadequately presented information, redundant content, construction and structure, insufficient supporting information, guiding issues, and layout and formatting, many of which were discussed by our participants as well (all but passive voice, construction and structure, insufficient supporting information, guiding issues). While Henriksen and colleagues conducted cognitive interviews to seek feedback on a questionnaire, the problems identified and solutions undertaken were similar to those in our study.

Participants in our study thought a website was an ideal method for delivering the MedManageSCI toolkit. Adults with SCI/D indicated that the benefits of a web-based toolkit in comparison to a paper-based toolkit included the potential of having a wider reach, being easier to update as new research or information is published, not having to worry about misplacing material, and having the ability to easily refer to it during appointments with their healthcare providers. Adults with SCI/D have previously reported the internet as a primary method for accessing information and have indicated a preference for programs and interventions to be web-based [40–45]. For example, Allin and colleagues used a participatory design process to develop SCI & U, a web-based, self-management program for persons with SCI/D in Canada [44]. This program focused on the management of diet, exercise, mental health, pain, bladder, bowel, sexuality, and skin. While medications may have been discussed within these topics, it was not the primary focus. SCI & U was then evaluated in a mixed methods pilot study and was found to be feasible and able to improve mood, resilience, self-efficacy, and secondary conditions, although these improvements were not statistically significant [46]. Ultimately, web-based interventions for self-management and behaviour change may have a positive impact on cognitive, emotional, and behavioural outcomes, while also supporting the scalability and cost-effectiveness of the intervention [47,48].

While web-based delivery has the potential to exclude individuals who do not have access to the internet, in Canada, approximately 94% of the population uses the internet, which is expected to increase to over 95% by 2029 [49]. Web-based interventions have the potential to reduce inequities by providing information in an accessible way and enabling patients to actively engage in their own healthcare [48]. However, access to the internet is only the first step, as it is also important to consider literacy, general health literacy, and eHealth literacy of the end-users to ensure individuals have the knowledge, skills, and abilities to seek, find, understand, and apply the information. A systematic review conducted by Silva and colleagues examined evidence on health literacy among individuals with SCI/D and found heterogenous results among the five included studies [50]. Adequate or reasonable health literacy was identified in three of the five studies (conducted in the United States) and inadequate or problematic health literacy was identified in one study (conducted in Turkey). The final study compared health literacy among racial groups. In a cross-sectional study conducted in Canada by Singh and colleagues, adults with SCI/D demonstrated moderate levels of eHealth literacy [51]. Furthermore, in the development of web-based tools, it is important to consider the literacy, health literacy, and eHealth literacy levels of the end-users, which is often recommended to be at the sixth grade level [52,53]. The use of visual aids, such as graphic symbols, multimedia, and pictograms, in health education materials has been shown to significantly improve the comprehension of information among those with lower literacy levels [54]. When using data visualization tools to support comprehension of information, it is important to ensure the message is clearly framed and use visual aids that align with the end-users’ preferences [55]. Additionally, the creation of comprehensible health educational materials can be supported by actively involving end-users throughout the development process.

Finally, participants in our study noted that some modules in the MedManageSCI toolkit would be more applicable to individuals who were newly injured or who were experiencing specific challenges with medication self-management. While not explicitly discussed by participants in our study, the MedManageSCI toolkit may also be more relevant to those who are more years post-SCI/D. As individuals with SCI/D age, they are more likely to experience secondary conditions such as high blood pressure, cardiac complications, and respiratory complications [56]. These conditions are often managed with prescription medications, meaning that individuals’ medication regimens may change, or become more complex, as they age. The applicability of MedManageSCI based on time post-injury may have implications on the implementation, dissemination, uptake, and sustainability of the toolkit as the timing of delivery needs to be considered. In a longitudinal study conducted by Matter and colleagues, the authors discussed the importance of individuals with SCI/D having access to timely, high-quality medical information post-discharge and throughout their lifetime to manage secondary conditions and to improve their health and quality of life [43]. The web-based delivery of MedManageSCI supports this point by Matter et al., as individuals with SCI/D will be able to access the toolkit at any timepoint post-injury. Furthermore, it will be important for individuals to be aware of and have access to the toolkit while in hospital and rehabilitation and to be able to refer to it while they age based on their medication information needs. The continued involvement of individuals with SCI/D in this research will support the implementation and sustainability of MedManageSCI [57–59].

### Future research

We believe the MedManageSCI toolkit can be useful for improving medication self-management among persons with SCI/D; however, more work is needed to understand its benefit. The next step of this research involves a mixed methods pilot study among persons with SCI/D to assess the feasibility, acceptability, and appropriateness of MedManageSCI. We will also assess the usability of MedManageSCI and its impact on beliefs about medications, medication self-efficacy, medication management capacity, and quality of life. If MedManageSCI is feasible, acceptable, appropriate, usable, and supports improvement in outcomes related to medication self-management, a key next step will be to explore barriers and facilitators to large-scale implementation and spread in real-world settings.

While many accessibility considerations were discussed and addressed during the co-design of MedManageSCI, it will be important to assess the accessibility of the toolkit on an ongoing basis to ensure it is meeting the needs of the end-users. Prior to making MedManageSCI publicly available, a member of our research team with expertise in accessibility and web-development reviewed the website according to the Web Content Accessibility Guidelines 2.1 to ensure the four principles of accessibility (perceivable, operable, understandable, robust) were met [60]. The purpose of these guidelines is to make online content more accessible to individuals with disabilities. Participants in our study recommended having the toolkit available in multiple languages to better address the medication management needs of individuals with SCI/D who do not speak English as their first language. For future research, the MedManageSCI toolkit could be translated to other languages using a translation/back-translation process, while also involving individuals with SCI/D [61]. Lastly, this research study focused on how adults with SCI/D found the clarity, comprehensiveness, relevance, and delivery of the MedManageSCI modules, while also exploring their thoughts on the design. Even though adults with SCI/D are the targeted end-users of this medication self-management toolkit, it can be accessed by care partners and healthcare providers. As such, it may be beneficial for future research to explore how care partners and healthcare providers understand the toolkit modules and their recommendations for improvement. MedManageSCI was designed specifically for adults with SCI/D in Canada; however, the processes and lessons learned might be of values to others who are developing web-based toolkit prototypes.

### Limitations

The toolkit was only available online and in English; therefore, it is possible that individuals within the SCI/D community were unable to participate if they did not have access to the internet or were not comfortable reading and communicating in English. Even with attempts to recruit participants from across Canada, we only had representation from four provinces, with the majority of participants living in Ontario. Despite this, Ontario is Canada’s largest province, with approximately 33,000 individuals living with SCI/D [62]. It is likely that most participants were from Ontario because that is where the majority of the research team is situated and has stronger social networks and recruitment ties. The overall sample size of 16 participants meant that several demographic subgroups had small numbers, which may limit transferability of this research. Given that the toolkit was developed among persons with SCI/D living in Canada, transferability to other health systems may need to be explored. The research team had prior relationships with some of the study participants due to their involvement in previous phases of this research. While we believe this strengthened the quality of the collected data, it is possible that the nature of these interviews differed from participants who did not have prior relationships with the research team. The interviews were not transcribed and therefore were not returned to participants for member checking; however, the interviewer ensured understanding during each interview by repeating and paraphrasing the participants recommendations for improvement. The usability of the toolkit was not assessed in this study as participants were guided by the interviewers; future research should explore its independent use across different devices. Finally, some recommendations made by participants could not be addressed due to feature limitations within the video software used (e.g., a recommendation was made to include more diversity in the level of injury by having some characters using a variety of mobility aids, (i.e., manual wheelchair, power wheelchair, walker); however, options were limited due to the software).

## Conclusions

Through cognitive interviews, adults with SCI/D provided recommendations to improve the comprehensibility and design of the toolkit. Revisions to the MedManageSCI prototype have been completed and the website is now publicly available (www.medmanagesci.ca). The findings from this study highlighted the positive perceptions of the web-based MedManageSCI toolkit among initial users. As a necessary next step, we plan to conduct further research to assess the feasibility, acceptability, appropriateness, and usability of MedManageSCI, along with outcomes related to medication self-management. Ongoing involvement of adults with SCI/D through the co-development and refinement will support implementation, uptake, and sustainability of MedManageSCI to ensure the toolkit is clear, comprehensive, relevant, and delivered in a way that meets individuals’ medication management needs.

## Supporting information

S1 File Standards for Reporting Qualitative Research (SRQR) checklist. (PDF)

S2 File Cognitive Interview Guide. (PDF)

S3 File Module Review Counts by Participant. (PDF)

S4 File Cognitive Interview Coding Matrix. (PDF)

S5 File Downloadable checklist of recommendations for web-based toolkit prototype development. (PDF)
