# Supplementary material for: A qualitative study to refine and finalize the MedManageSCI prototype: A web-based toolkit to support medication self-management in adults with spinal cord injury/dysfunction
Source: PLOS Digit Health. 2025 Oct 22;4(10):e0001054. doi: 10.1371/journal.pdig.0001054 (PMC12543128; doi:10.1371/journal.pdig.0001054)
Supplement: S2 File — (PDF) [file pdig.0001054.s002.pdf]

Cognitive Interview Guide

Introduction & Background

1. I'd like to start by learning a bit about you. Would you please tell me about yourself and your journey with a spinal cord injury?

SECTIONS

1. What thoughts came to mind while reading [section]?
2. Can you explain to me in your own words what [section] described?
3. Are there any words that you did not understand or that others may not understand?
4. What thoughts did you have about the length of module [#]?
    a. Is there any content that you think should be removed?
5. Do you think that this [module] of the toolkit will be useful?
    a. Why or why not?
    b. What would you use it for?

DESIGN AND GRAPHICS

1. What was your overall impression of the toolkit design?
    a. How visually appealing did you find the toolkit?
    b. What did you like? Why? Not like? Why?
    c. What could make it better? Other visuals?
2. What thoughts did you have about the [design/ layout]?
3. What thoughts did you have about the colours and contrast [of the infographic/ video]?
4. What thoughts came to mind while looking at [infographic, video, picture]?

OTHER

1. Would you recommend the toolkit to someone with a spinal cord injury?
    a. *Why or why not?*
2. What suggestions do you have for changing the toolkit so it is easier to use?
3. The toolkit was offered on [website]. How did you feel about accessing the toolkit in this way?
    a. *What other delivery options are there?*
    b. *Would those options be preferred over the [website]?*
4. We are almost done our interview. Is there anything else that you would like to share about the toolkit?

End of Interview
