# Supplementary material for: A qualitative study to refine and finalize the MedManageSCI prototype: A web-based toolkit to support medication self-management in adults with spinal cord injury/dysfunction
Source: PLOS Digit Health. 2025 Oct 22;4(10):e0001054. doi: 10.1371/journal.pdig.0001054 (PMC12543128; doi:10.1371/journal.pdig.0001054)
Supplement: S3 File — (PDF) [file pdig.0001054.s003.pdf]

Module Review Counts by Participant

	Module #								
	1	2	3	4	5	6	7	8	9
**Participant 1**	✓	O							
**Participant 2**	✓	O	✓						
**Participant 3**	✓			✓					
**Participant 4**	✓				✓			✓	
**Participant 5**	✓					O			
**Participant 6**									✓
**Participant 7**							O		
**Participant 8**	✓					✓	✓		
**Participant 9**	✓		✓						
**Participant 10**	✓								✓
**Participant 11**		✓						✓	
**Participant 12**		O							
**Participant 13**					✓				
**Participant 14**						O			
**Participant 15**						✓			
**Participant 16**				✓			✓		

✓ Full module; O Part of module
