# Supplementary material for: A qualitative study to refine and finalize the MedManageSCI prototype: A web-based toolkit to support medication self-management in adults with spinal cord injury/dysfunction
Source: PLOS Digit Health. 2025 Oct 22;4(10):e0001054. doi: 10.1371/journal.pdig.0001054 (PMC12543128; doi:10.1371/journal.pdig.0001054)
Supplement: S4 File — (PDF) [file pdig.0001054.s004.pdf]

Cognitive Interview Coding Matrix

Part 1:

Module #			
Module Section			
Participant ID			
**Sections**			
1. What thoughts came to mind while reading [section]?			
2. Can you explain to me in your own words what [section] described?			
3. Are there any words that you did not understand or that others may not understand?			
4. What thoughts did you have about the length of module [#]?<br>a. Is there any content that you think should be removed?			
5. Do you think that this [module] of the toolkit will be useful?<br>a. Why or why not?<br>b. What would you use it for?			
**Section – Design and Graphics**			
3. What thoughts did you have about the colours and contrast [of the infographic/ video]?			
4. What thoughts came to mind while looking at [infographic, video, picture]?			
**Overall – Design and Graphics**			
1. What was your overall impression of the toolkit design?<br>a. How visually appealing did you find the toolkit?<br>b. What did you like? Why? Not like? Why?<br>c. What could make it better? Other visuals?			
2. What thoughts did you have about the [design/ layout]?			
**Other**			
1. Would you recommend the toolkit to someone with a spinal cord injury?<br>a. Why or why not?			
2. What suggestions do you have for changing the toolkit so it is easier to use?			
3. The toolkit was offered on [website]. How did you feel about accessing the toolkit in this way?<br>a. What other delivery options are there?<br>b. Would those options be preferred over the [website]?			
4. We are almost done our interview. Is there anything else that you would like to share about the toolkit?			

Part 2:

Module	Section	Type of Revision	Description of Revision	Completed	Person Responsible	Notes
