# Supplementary material for: A qualitative study to refine and finalize the MedManageSCI prototype: A web-based toolkit to support medication self-management in adults with spinal cord injury/dysfunction
Source: PLOS Digit Health. 2025 Oct 22;4(10):e0001054. doi: 10.1371/journal.pdig.0001054 (PMC12543128; doi:10.1371/journal.pdig.0001054)
Supplement: S5 File — (PDF) [file pdig.0001054.s005.pdf]

Checklist of recommendations for web-based toolkits

☑	**Recommendations for web-based toolkits**
☐	Complete multiple rounds of revisions to the written content:<br>• Research team members<br>• Experts in the field outside of the direct research team<br>• End-users<br>• Other individuals not directly involved in the creation of the content
☐	Read written content out loud to team members or end-users to catch repetitive words or phrases
☐	Use headings and subheadings to organize the information on the website pages and within the modules
☐	Add bullets and spacing to break-up larger sections of text
☐	Use accessible language and check literacy levels of the written content:<br>• If literacy level is high (based on readability scores), add practical examples and visuals (e.g., infographics, videos) to supplement the written information
☐	Add a glossary so frequently used words and complex terms or phrases have a definition linked to them
☐	Emphasize key points through formatting – add icons, bold and/or underline words
☐	Include direct links to references and other similar or relevant resources
☐	Ensure all downloadable resources are fillable (e.g., they can be completed digitally on a computer, tablet, or mobile phone, or they can be printed and written on)
☐	Use line-by-line colour coding in larger tables (e.g., those with multiple columns and rows)
☐	Use colour contrast checkers to ensure colour combinations meet accessibility requirements
☐	Involve end-users in the selection of pictures/images for the toolkit to ensure all pictures are representative of the population
☐	Include diversity in the selected images (e.g., differing ages, genders, levels of injury, types of mobility devices)
☐	Include alternative text for all pictures
☐	Ensure the website meets web accessibility standards
☐	Use a design that allows for the creation of a paper-based version
☐	Consider having the information available in multiple languages
☐	Seek ongoing feedback from the end-users throughout the process
